# Drill Holes and Predation Traces versus Abrasion-Induced Artifacts Revealed by Tumbling Experiments

**DOI:** 10.1371/journal.pone.0058528

**Published:** 2013-03-07

**Authors:** Przemysław Gorzelak, Mariusz A. Salamon, Dawid Trzęsiok, Robert Niedźwiedzki

**Affiliations:** 1 Department of Biogeology, Institute of Paleobiology, Polish Academy of Sciences, Warsaw, Poland; 2 Department of Palaeontology and Biostratigraphy, Faculty of Earth Sciences, University of Silesia, Sosnowiec, Poland; 3 Institute of Geological Sciences, Wrocław University, Wrocław, Poland; University of Florence, Italy

## Abstract

Drill holes made by predators in prey shells are widely considered to be the most unambiguous bodies of evidence of predator-prey interactions in the fossil record. However, recognition of traces of predatory origin from those formed by abiotic factors still waits for a rigorous evaluation as a prerequisite to ascertain predation intensity through geologic time and to test macroevolutionary patterns. New experimental data from tumbling various extant shells demonstrate that abrasion may leave holes strongly resembling the traces produced by drilling predators. They typically represent singular, circular to oval penetrations perpendicular to the shell surface. These data provide an alternative explanation to the drilling predation hypothesis for the origin of holes recorded in fossil shells. Although various non-morphological criteria (evaluation of holes for non-random distribution) and morphometric studies (quantification of the drill hole shape) have been employed to separate biological from abiotic traces, these are probably insufficient to exclude abrasion artifacts, consequently leading to overestimate predation intensity. As a result, from now on, we must adopt more rigorous criteria to appropriately distinguish abrasion artifacts from drill holes, such as microstructural identification of micro-rasping traces.

## Introduction

Predator–prey interaction is one of the key systems to understand the evolution of organisms in both modern and past ecosystems [1]–[6]. However, the role of predation in evolution is hard to evaluate accurately in the fossil record. Predation traces, such as drill holes, are one of the most powerful and widely used proxies for predation intensity since they provide direct evidence of predator-prey interactions [3]. These traces have been commonly used to document various predation patterns supporting dramatic changes in the fossil marine ecosystem record, such as the Middle Paleozoic Marine Revolution (MPMR) and Mesozoic Marine Revolution (MMR) [2], [7]–[11].

As far as a huge body of literature describe both recent and fossil drill holes [2], [3], [8]–[16], their recognition and verification of predatory origin can still be problematic despite a wide array of, both qualitative and quantitative, criteria [3], [17]. Actually, properly distinguishing traces produced by drilling predators from those produced by other biotic and abiotic factors (including parasitism, dissolution, abrasion or bioerosion) remains hard to tell in practice [18]–[21]. Consequently, traces other than predatory drillings can be misidentified and erroneously treated as holes of predatory origin, inducing overestimation of predation pressure.

In the following, we intend to reconsider the origin of holes commonly recorded in the fossil shells and provide evidence that abrasion may leave holes strongly resembling drilling-predator traces.

## Materials and Methods

To simulate shell deterioration/abrasion in seawater-agitated environment, three independent tumbling experiments using a rotating barrel LPM-20 (Glass GmbH & Co. KG Spezialmaschinen) were performed at the Faculty of Earth Sciences, Laboratory of Palaeontology & Biostratigraphy of the University of Silesia. No specific permissions were required for performing these experiments. We tested commercially available shells of extant unionid bivalves (Unionidae indet.), gastropods (*Nassarius* sp.) and brachiopods (*Frenulina sanguinolenta*) with smooth margins and non-abraded, intact surfaces. These shells were tumbled at 30 revolutions per minute [rpm] for 1 h, 2 h, and 4 h respectively in a barrel containing 1 kg quartz gravels (ca. 20 mm in diameter), 0.3 kg medium-size sand and 3L of artificial sea water. Given a tumbling barrel with a 27-cm-diameter and the rotation speed of 30 [rpm], the tumbling speed approximates wave-action of 0.135 m/s. One hour of tumbling is thus time equivalent to ca. 0.5 km of transport or in-place tumbling within the surf zone. After each of the three tumbling periods, shells were removed from the barrel and examined for any potential damage to the shell.

The inner outlines of holes were drawn using a camera lucida. In some cases, where possible, the approximate geometrical shape of holes in vertical cross sections was determined by making a plasticine mold. Measurements were made using electronic calipers. The data was analyzed using PAST 2.02 software [22]. Observations of selected specimens were conducted with Scanning Electron Microscope Philips XL−20 at the Institute of Paleobiology of the Polish Academy of Sciences in Warsaw.

The specimen collection is housed at the Department of Palaeontology and Biostratigraphy of the University of Silesia, Sosnowiec, Poland (catalogue number GIUS 12-3616– Geological Institute of the University of Silesia).

## Results

**Figure 1:**
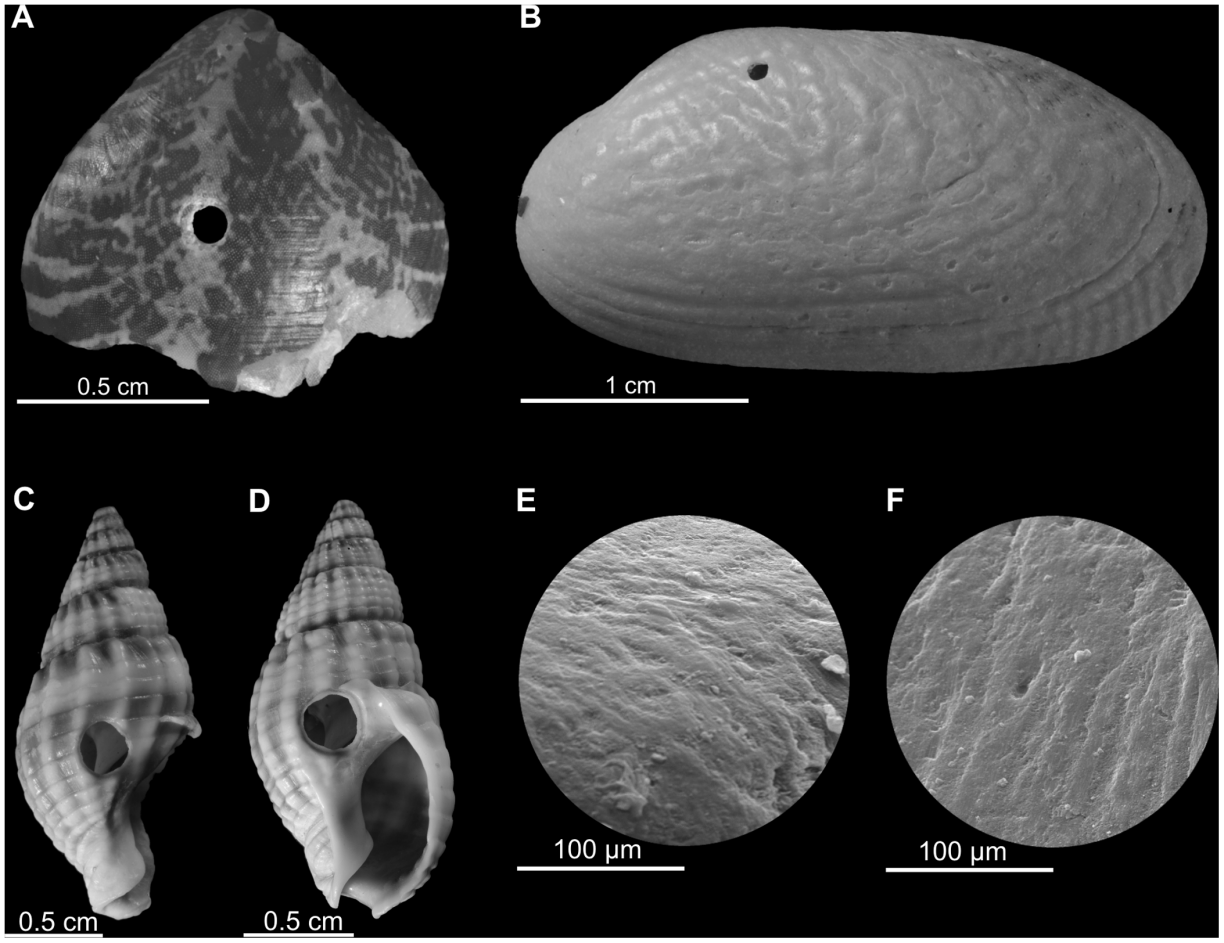
Holes generated by tumbling experiments on various shells. (**A**) Brachiopod shell (*Frenulina sanguinolenta*) (GIUS 12-3616/Fs1) after 4 hours of tumbling. (**B**) Unionidae bivalve shell (GIUS 12-3616/U1) after 1 hour of tumbling. (**C–D**) Gastropod shells (*Nassarius* sp.) (GIUS 12-3616/N1-2) after 2 hours of tumbling. (**E–F**) Close up of hole margins in *Nassarius* sp.

**Figure 2:**
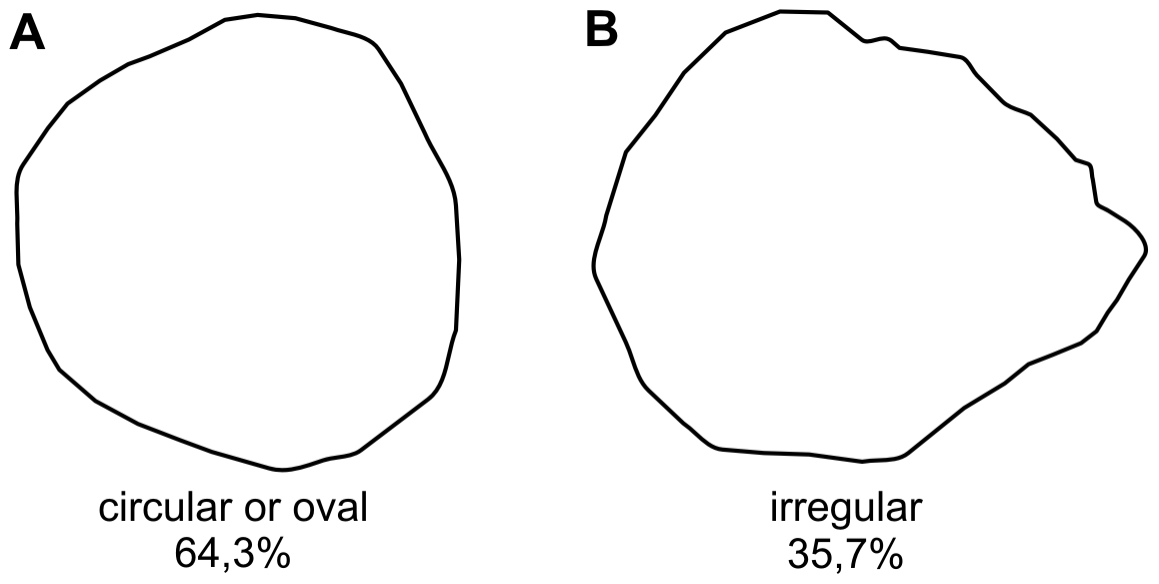
Two morphotypes of the inner outlines of holes and their frequency distribution (drawings by camera lucida).

**Figure 3:**
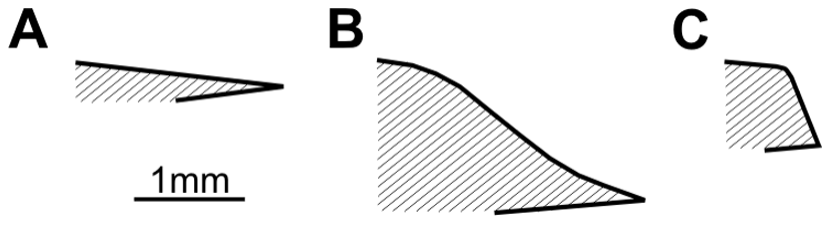
Projections of hole margins at vertical cross sections.

**Figure 4:**
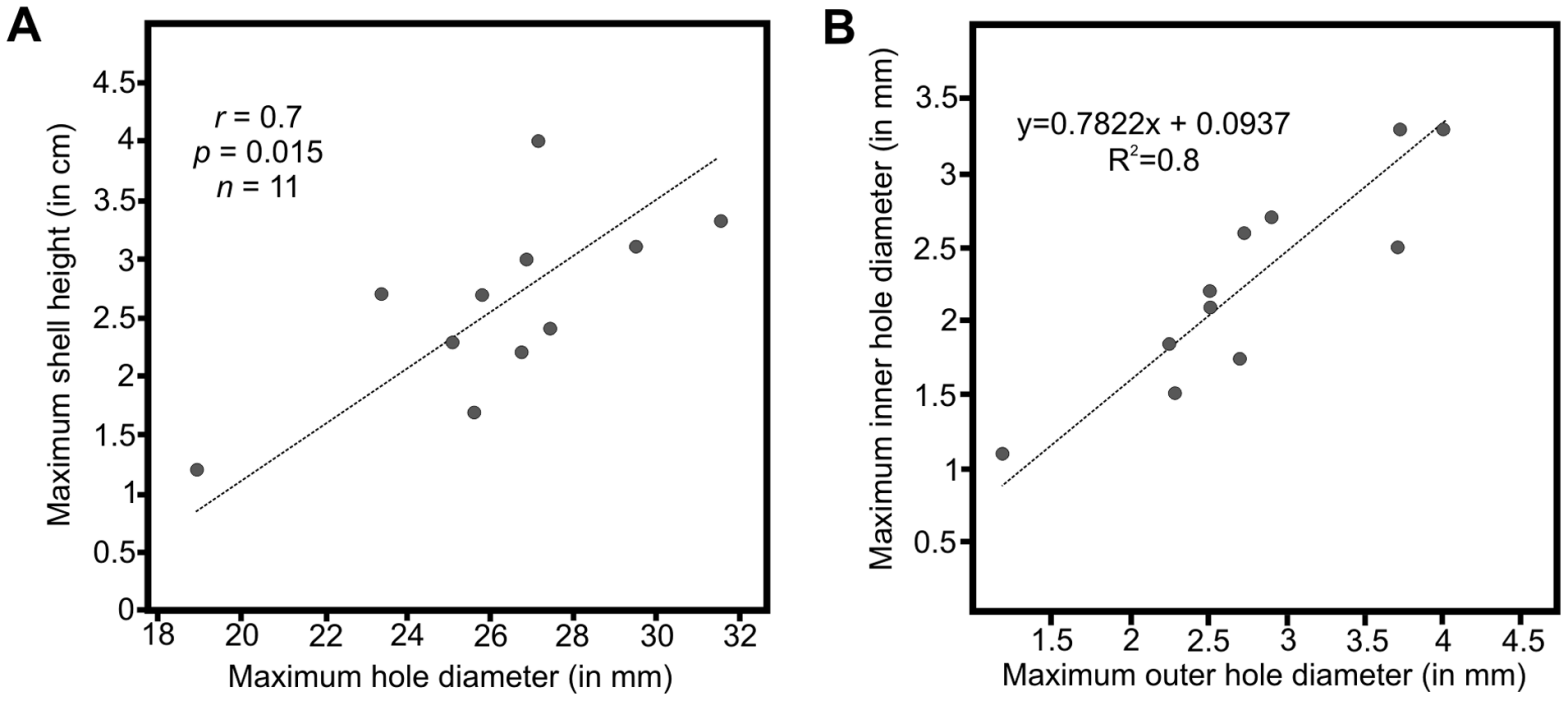
Scatter plots showing two types of correlations. (**A**) Correlation between hole size and shell size. (**B**) Correlation between the inner and outer hole diameter in *Nassarius* sp. Symbols for (A): *r*–Spearman rank correlation, *p*–probability of Type I Error for H [0]: *r* = 0, *n*–sample size.

After each tumbling experiment, shells were only slightly abraded and not significantly damaged. However, in some cases, tumbling induced small singular (only rarely multiple) holes that completely penetrate the shells. These holes are circular, oval or irregular and perpendicular to the shell surface (Figure 1, Figure 2). The majority of holes are smooth although some display an irregular outline. Their vertical cross sections are commonly parabolic or plane but inclined at different angles with smaller inner hole openings than outer ones (Table 1, Figure 3). There is a significant positive correlation between shell size and hole diameter at least for gastropods (Figure 4A). Similarly, there is also a strong correlation between the outer and inner hole diameters (Figure 4B).

**Table 1:** Results of tumbling experiments on various shells.

Investigated taxa	Number of shells	Duration of experiment (in hours)	Frequency of singular holes(in %)	Size range of shells with holes (in cm)	*Holes*			
					Range of maximumouter diameter (in mm)	Range of minimum outer diameter (in mm)	Range of maximum inner diameter (in mm)	Range of ratio of inner to outer diameter
*Nassarius* sp.	19	2	57.9	1.9–3.15	1.2–4	0.9–3.8	1.1–3.3	0.6–0.9
Unionidae indet.	8	1	25	1.8–3.2	0.8–1.2	0.7–1.0	0.7–1.1	0.9
*Frenulina sanguinolenta*	2	4	50	1.6	1.5	1.1	0.8	0.53

**Figure 5:**
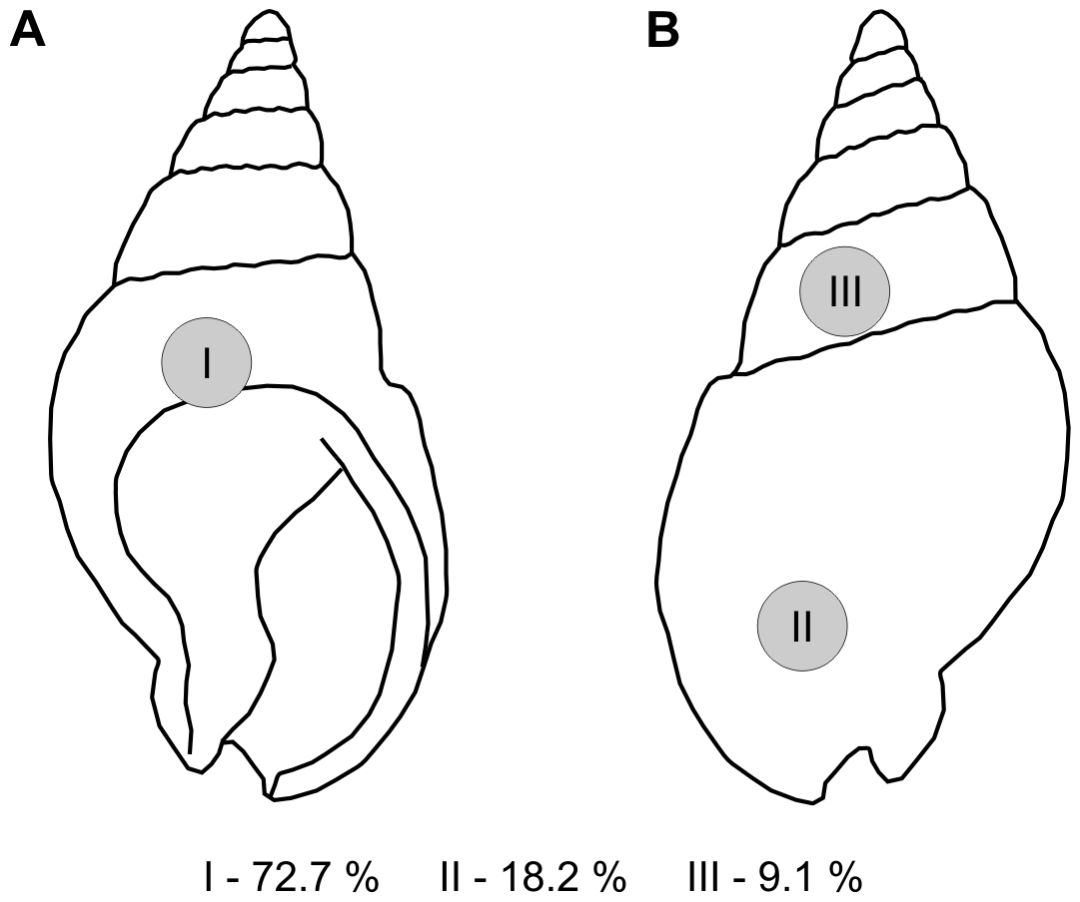
Frequency distribution of holes in *Nassarius* sp. (**A**) Apertural view. (**B**) Abapertural view.

Furthermore, gastropod shells display non-randomly distributed holes (Figure 5), i.e., these latter are predominantly located near their aperture.

## Discussion

Whether tumbling experiments imitate accurately the natural tumbling conditions experienced by shells in the surf zone has been argued [23]–[25]. However, such experiments certainly may provide valuable insights into the character of mechanical damage and abrasion generated at the shell surface by abiotic processes [26]–[28]. Our tumbling experiments clearly showed that abrasion-induced holes can be important taphonomic process. If these artifacts are not identified appropriately or accurately, this can lead to an overestimation of predation intensity in the fossil record.

In recent years, numerous lines of criteria have been proposed to recognize predatory drill holes. These can be separated into two groups: (i) non-morphological criteria, i.e., evaluation of holes for non-random (site-specific, size-selective, or taxon restricted distribution of traces) [29]–[34] and (ii) morphometric criteria, i.e., quantification of drill-hole shapes and their size [8], [35]–[38].

Predatory borings are generally defined as commonly single and unhealed perforations perpendicular to the valve surface, having circular to oval shapes, and regular outlines although irregular shapes and outlines have been also noted (compare figure 4 in [36]). Furthermore, the ratio of inner to outer diameter commonly exceeds 0.5 [8]. Correlation between size of holes and size of bored fossils has been also used to support the predatory origin of such traces [32].

Although considerable effort has been devoted to establish the reliable identification criteria for predation traces, the present data from tumbling experiments suggest that the existing methods are insufficient to exclude abrasion artifacts. Nearly all features of holes generated on shell surfaces during our tumbling experiments (including shape, outline, ratio of inner to outer diameter, correlation between size of holes and size of shells as well as between the inner and outer hole diameter; see Figure 1, Figure 4 and Table 1) are identical to those observed in recent and fossil holes commonly ascribed to drilling predation (compare figure 1 in [8]; figure 1I, and 2B,G,K,N,P in [39]).

An accurate identification of the underlying causes of these surprising results, especially for a site-specificity, is difficult. It can be speculated, however, that at least two mechanisms may be involved in the observed pattern. First, the holes may have developed as a consequence of a progressive and preferential abrasion experienced by the knobs of apertural side of a gastropod shell in a rotating barrel leading to the directional thinning of the specific site of the shell. Alternatively, such holes may be formed *via* the preferential hits of the apertural side of a gastropod shell in a rotating barrel by pebbles leading to the formation of perforation due to the weakening of the shell structure in that area. Then, more or less regular shape of such holes may be modeled with suspended sediment in an agitated seawater.

Given above, making a reliable estimation of predation intensity in the fossil record seems very difficult. Considering the utility of drill holes as predation proxies, novel techniques for reliable identification of predatory traces are needed. Recently developed microstructural analyses (such as the identification of radular rasping marks on drill-hole walls) provide the most promising criteria to accurately identify the drillers [39], [40]. However, we have to keep in mind that the drilling process, when chemically aided, may sometimes obliterate such predatory microtraces. Furthermore, abrasion may also wear away microstructural details of the surface texture or even possibly produce shallow grooves (wear scars) which may seemingly mimic radular rasping marks (compare figure 3A in [41]).
